# Effects of combined trunk stretching and lumbar stabilization exercises for chronic non-specific low back pain: a randomized clinical trial

**DOI:** 10.1590/1414-431X2025e14863

**Published:** 2026-03-09

**Authors:** C.C.C. Coutinho, D.D.A. Sampaio, N.D.C. Pereira, A.C. Pássaro, V.M.A. Oliveira, R.A. Casarotto

**Affiliations:** 1 Departamento de Fisioterapia, Universidade Federal da Paraíba, João Pessoa, PB, Brasil; 2 Departamento de Fisioterapia, Fonoaudiologia e Terapia Ocupacional, Faculdade de Medicina, Universidade de São Paulo, São Paulo, SP, Brasil

**Keywords:** Exercise therapy, Low back pain, Physiotherapy, Rehabilitation, Muscle stretching

## Abstract

This study aimed to compare the effectiveness of combining trunk stretching with lumbar segmental stabilization versus exclusively performing lumbar segmental stabilization exercises for treating chronic nonspecific low back pain (CNLBP). Thirty-four CNLBP subjects were randomized into two groups: active trunk stretching + lumbar segmental stabilization (AS+LSS, n=17) and placebo stretching + lumbar segmental stabilization (PS+LSS, n=17). One-hour sessions were performed twice a week for six weeks. Pain intensity, pain quality, functional disability, global impression of recovery, emotional state, symptoms, and adverse effects were assessed at baseline, after 6 weeks, and at 12 and 24 weeks follow-up. Both groups experienced significant reductions in pain intensity and functional disability after the intervention. Depression and anxiety showed significant improvements during the intervention but did not persist at follow-up. No statistically significant differences were observed between the groups for the studied variables. The study concluded that both protocols are beneficial for CNLBP patients, suggesting that lumbar segmental stabilization exercises alone are sufficient for reducing pain and functional disability.

## Introduction

Chronic nonspecific low back pain (CNLBP) is defined as pain and discomfort localized in the lumbosacral region, without specific underlying pathological causes persisting for more than 3 months. It is frequently associated with functional, emotional, and social impairments (1), which are linked to significant clinical and economic burdens (2). Research indicates that the number of individuals with disability caused by CNLBP has increased by 54% over the past 30 years, with the total treatment cost estimated to exceed 100 billion US dollars annually in the United States (2).

A widely discussed mechanism in CNLBP is lumbar spine instability (3) and impairment in the control of lumbopelvic muscles, such as the transversus abdominis and multifidus, a characteristic present in patients with low back pain (LBP), especially in those with chronic symptoms (4). However, the reason for this impaired neuromuscular control is not yet fully understood (5).

Clinical practice guidelines recommend exercise for CNLBP (6), with lumbar segmental stabilization (LSS) showing beneficial effects on pain, function, and recurrence prevention by improving motor control and the endurance of stabilizing muscles (7,8). On the other hand, trunk stretching has shown benefits in these patients, as there is a positive correlation between reduced trunk flexibility and low back pain symptoms, evidenced by reduced lumbar spine flexibility in the frontal, transverse, and sagittal planes (9). However, there is still limited evidence to support the use of a specific type of exercise or therapeutic program over another (10). Recent findings suggest that many clinical trials may prescribe exercise below the minimum effective dose recommended by the World Health Organization, which may contribute to inconsistent outcomes (11). These therapeutic strategies are based on the rationale of restoring spinal stability and mobility through specific motor control and stretching interventions (12).

Clinical trials have investigated the combination of LSS with muscular stretching to enhance outcomes in chronic nonspecific low back pain (13,14). However, current evidence suggests that this combination is not superior to LSS alone in reducing pain intensity in short-term protocols (13). This may be because intervention durations are often limited to six or eight weeks, which appear insufficient to induce meaningful neuromuscular adaptations and flexibility gains (13,15). A recent meta-regression reinforces that exercise effectiveness depends on adequate frequency and duration, with optimal outcomes occurring when protocols meet minimum thresholds for time and dose (16). Supporting this, Plandowska et al. (17) observed that an eight-week program combining core stabilization exercises and active hamstring stretching produced significant improvements in pain and hamstring flexibility in young adults with nonspecific low back pain, with changes becoming evident after the full intervention period. Therefore, the effects of combining stretching and LSS remain uncertain, especially when applied over short durations.

A recent systematic review and meta-analysis highlighted that most clinical trials evaluating the efficacy of specific exercises for nonspecific low back pain in the general population were of low methodological quality, with 85.2% classified as low quality and only 14.8% as moderate. The overall certainty of the evidence was rated as very low to low, mainly due to short intervention durations and inadequate follow-up (18).

Furthermore, following the recommendations of the IMMPACT (Initiative on Methods, Measurement and Pain Assessment in Clinical Trials) group for the multidimensional assessment of chronic pain, our line of study incorporates psychological variables, such as depression and anxiety (19). The inclusion of these variables is justified by the strong association between chronic low back pain and depressive and anxiety symptoms, which may negatively influence the response to physical treatment, and by findings that therapeutic exercise can promote emotional well-being and enhance clinical outcomes in chronic pain populations (20).

Considering the above, we hypothesized that adding stretching to LSS offers complementary improvements in physical and psychological outcomes in adults with CNLBP. Physiologically, this combination may enhance flexibility and joint mobility, reduce compensatory movements, and promote neuromuscular activation (12,21). While stabilization exercises primarily target deep motor control and segmental coordination (12), stretching programs - such as those based on global postural reeducation - have demonstrated positive effects on flexibility and functional improvement in chronic low back pain (21).

Thus, this study aimed to verify whether combining trunk stretching with LSS is comparable to exclusively performing LSS exercises in improving pain, functional limitation, depression, anxiety, and overall treatment perception in CNLBP. Therefore, our results may guide physiotherapists in selecting the most effective exercises for individuals with CNLBP.

## Material and Methods

This randomized trial, registered with the number NCT02985892, was conducted according to the Consolidated Standards of Reporting Trials (CONSORT) and Template for Intervention Description and Replication (TIDieR) guidelines. The study protocol was approved by the Ethics Committee of the School of Medicine, University of São Paulo (Protocol No. 096/16; CAAE 54696616.6.0000.0065) and was conducted according to the Declaration of Helsinki. All participants eligible for the study were informed of the research procedures, and those who agreed to participate provided written informed consent, according to Resolution 466/2012 (Brazilian National Health Council).

**Figure 1:**
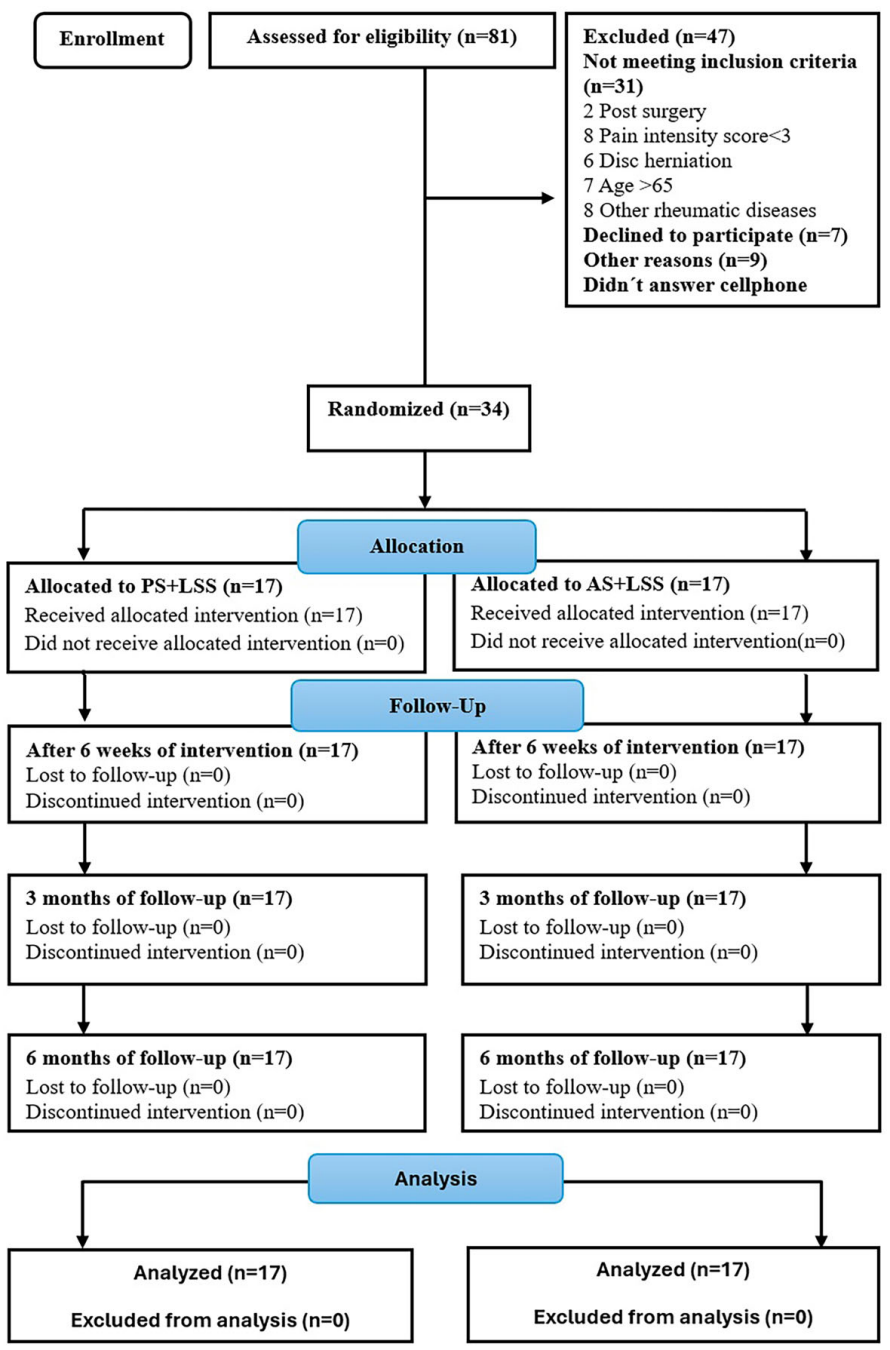
Flowchart of study design. PS+LSS: placebo stretching + lumbar segmental stabilization; AS+LSS: active trunk stretching + lumbar segmental stabilization.

Participants with CNLBP were recruited at the Physiotherapy Teaching Clinic of the Federal University of Paraiba. Blinded examiners determined whether patients were eligible to participate in the study based on clinical history and examination (Figure 1). Inclusion criteria were: 1) diagnosis of CNLBP, defined as pain or discomfort between costal margins and inferior gluteal folds without referred lower limb pain; 2) pain intensity of at least three points in the Numeric Pain Rating Scale (NPRS); 3) persistent LBP for at least 3 months; 4) any gender; and 5) aged between 18 and 65 years. Exclusion criteria were: 1) spine or shoulder degenerative or inflammatory pathologies; 2) episodes of acute shoulder pain; 3) vertebral column tumors; 4) nonunion or malunion of spine, shoulder, or upper limb fractures; 5) recent spine, shoulder, or upper limb surgeries; 6) herniated disc; 7) spondylolysis or spondylolisthesis; 8) any rheumatic diseases; 9) involvement in workplace lawsuit; 10) undergoing any other physical therapy treatment or taking pain relief medication.

The intervention was conducted by a physical therapist with over 15 years of clinical experience in musculoskeletal rehabilitation and specific training in LSS. This professional was blinded to the participants' health conditions. To ensure consistency, all sessions followed a standardized protocol.

The sample size was calculated considering a statistical power of 80%, minimal important difference, standard deviation of two points, and 20% improvement on the pain numerical rating scale. Seventeen patients were required per group, considering a 5% significance level and 15% sample loss. This strategy is consistent with the CONSORT guidelines, which recommend that sample size calculations be based on the primary outcome to ensure sufficient power for the main hypothesis of interest, while secondary outcomes can enrich the interpretation but are not used as the basis for power estimation.

The participants were randomly allocated to active trunk stretching + lumbar segmental stabilization group (AS+LSS) or placebo stretching + lumbar segmental stabilization group (PS+LSS) by a blinded examiner not involved in other research procedures. Randomization was generated using a randomization website (www.randomization.com). The allocation was confidential, sealed in opaque envelopes, and numbered sequentially. Participants were also blinded to their treatment group.

### Intervention

Both intervention programs (AS+LSS and PS+LSS) consisted of 12 sessions of 60 min each over six weeks (i.e., two sessions per week). Interventions were performed by an experienced physiotherapist not involved in any other research procedure. Intervention specifications are described below (Table 1).

**Table 1:** Treatment protocol in active trunk stretching + lumbar segmental stabilization group (AS+LSS) and placebo stretching + lumbar segmental stabilization group (PS+LSS).

Group	Exercise	Position	Sets/Duration
AS+LSS	Active trunk stretching	Stretching of the pectoral and latissimus dorsi muscles while standing; performing complete shoulder abduction; bringing the hands together and stretching upward	3 sets, lasting 30 s each 30-s interval between exercises
		Stretching of the latissimus dorsi, teres major, rectus abdominis, and external oblique muscles; performing complete shoulder abduction; leaning sideways to the right, then to the left	
		Stretching of the internal and external obliques in lateral decubitus, leaning backward, keeping the pelvis fixed in position; performing the same movement on both sides	
PS+LSS	Placebo wrist stretching	Position stretching of wrist flexors while standingPosition stretching of the wrist extensors while standingPosition stretching of the wrist flexors; Partial wrist stretch.	3 sets of 30 s each 30-s interval between exercises
AS+LSS and PS+LSS	Lumbar segmental stabilization	Exercises for the lumbar multifidus (LM) in ventral decubitus Exercises for the transversus abdominis (TrA) in dorsal decubitus with flexed knees Exercises for the TrA in 4-point kneeling Co-contraction of the TrA and LM in upright position	4 sets of 10 repetitions Each contraction lasts 10 s 10-s interval between contractions 1-min interval between sets

AS+LSS: active trunk stretching + lumbar segmental stabilization group; PS+LSS: placebo stretching + lumbar segmental stabilization group.

The participants of the AS+LSS group performed three active trunk stretching exercises (involving rectus abdominis, internal and external oblique, latissimus dorsi, and teres major muscles) and lumbar segmental stabilization. The participants of the PS+LSS group performed only wrist flexion and extension exercises and lumbar segmental stabilization. Trunk stretching (AS+LSS group) and wrist exercises (PS+LSS group) were repeated three times for 30 s each, totaling 15 min. To minimize attention and performance bias, a placebo stretching condition was included to equalize session time and therapist interaction across groups. Wrist flexion and extension exercises were adopted as neuromuscularly neutral controls, following the rationale used in previous studies where distal motor tasks unrelated to the lumbar region were selected to avoid influencing target-area pain responses (22).

Both groups then performed four types of lumbar stabilization exercises targeting multifidus and transversus abdominis according to Richardson et al. (12) and Hodges and Richardson (4). These stabilization exercises were performed in four sets of 10 repetitions for 10 s each, with a 10-s rest between repetitions and 1-min interval between sets (Table 1).

The participants were instructed to report any complaints related or unrelated to the exercises, maintain their regular routines, and refrain from participating in other treatment programs.

### Outcomes

All instruments were selected according to IMMPACT recommendations for chronic pain treatment efficiency and efficacy (19). The main IMMPACT recommendations included in this clinical trial cover six domains: 1) pain; 2) physical function; 3) emotional aspects; 4) global impression of improvement; 5) symptoms and adverse events; and 6) patient satisfaction. Outcomes were measured before interventions (baseline), immediately after 6 weeks of intervention (discharge), and after 12 and 24 weeks of follow-up. Sociodemographic characteristics (i.e., age, weight, height, body mass index, marital status, and education level) were assessed at baseline.

#### Primary outcomes

Pain was assessed using the NPRS and the McGill pain questionnaire (MPQ) (23,24).

The NPRS scale is an 11-point numeric scale, which ranges from “0” (no pain) to “10” (worst pain imaginable), and it was used to measure the mean pain intensity of the patients over the last 7 days.

The MPQ comprises 78 pain descriptors categorized into four main classes (sensory, affective, evaluative, and miscellaneous), along with 20 subclasses. Each subclass consists of at least two and at most six words, with assigned intensity values. The Brazilian Portuguese adaptation and validation of the MPQ were conducted by Varoli and Pedrazzi (24). This instrument allows for a multidimensional assessment of pain, capturing not only its intensity but also the qualitative aspects through the patient's selection of descriptors that best characterize their pain experience. This qualitative profiling enables a more comprehensive understanding of how the pain is perceived, including its sensory and emotional components, which is particularly useful for evaluating the effects of therapeutic interventions on the nature of pain.

#### Secondary outcomes

Functional disability was estimated by the Roland-Morris Disability Questionnaire (RMDQ) (25), and emotional status was evaluated using Beck Depression Inventory (BDI) (26) and Global Anxiety-Visual Analog Scale (GA-VAS) (27).

The RMDQ contains 24 questions about daily life functioning. Each affirmative response corresponds to a one-point score, which summed provides a total score. A total score close to zero indicates none or little limitation, while a total score of >14 indicates significant limitation due to lumbar pain (25).

The BDI (score ranges from 0 to 63) is a self-reported questionnaire containing 21 questions scored from 0 to 3 points, and higher scores represent the worst depressive symptoms (26).

The GA-VAS assesses anxiety using a 100-mm horizontal scale (“not at all anxious” on the left end to “extremely anxious” on the right end) (27). Another secondary outcome was patient satisfaction, assessed after treatment using MedRisk (28) and Global Perceived Effect (GPE) scale (29).

The MedRisk instrument consists of 20 items (aspects of the health service distinct to patient-therapist interaction), and the satisfaction level of each item follows a 5-point Likert scale (“strongly disagree” to “strongly agree” or “not applicable”). Higher scores represent a high level of satisfaction.

The 11-point GPE scale (score ranges from -5 “vastly worse” to +5 “completely recovered”) evaluates global perception of change after receiving treatment by comparing symptoms at treatment initiation and current status, where higher scores are related to better impression of recovery.

MedRisk was assessed at discharge, while GPE was assessed at discharge and after 12 and 24 weeks of follow-up. Treatment adherence was assessed by recording the number of treatment sessions attended. At each session, patients were verbally asked how they were feeling, as well as about any use of analgesic medications or involvement in other forms of treatment. Additionally, participants were instructed to report any symptoms or adverse events, whether or not related to the intervention.

To minimize misunderstandings or assessment errors, an anonymous examiner read the questions of all instruments to the participants and transcribed their responses.

### Statistical analysis

Statistical procedures were performed according to intention-to-treat principles. First, we conducted descriptive analyses, visually inspected histograms, and used the Shapiro-Wilk test to verify data normality. Between-group differences adjusted for baseline and 95% confidence intervals (95%CI) were calculated using mixed linear models of group, time, and group-*vs*-time interaction. A first-order autoregressive covariance matrix (AR(1)) was selected, as it assumes that measurements taken closer in time are more correlated - appropriate for our repeated-measures design with regular intervals. Other structures, including identity and compound symmetry, were tested, but AR(1) provided a better model fit based on Akaike (AIC) and Bayesian (BIC) information criteria. Group and time factors were considered fixed factors and participants were considered random factors. A Q-Q plot was performed for all variables to confirm normality of residuals between groups. We used SPSS software version 20.0 (IBM SPSS Corp., USA) for all data analyses. Effect sizes and statistical significance are reported as mean difference (MD) and 95%CI (30).

Based on results for LBP improvement, minimal important differences adopted were 2.5 for PNRS, ≥3.5 for RMDQ, 6.9 for BDI, and the terms “much improved” or “very satisfied” for the GPE scale (29).

## Results

Thirty-four patients were included and completed all phases of the research, totaling 408 appointments, showing high adherence to the exercise programs.

The characteristics of the AS+LSS and PS+LSS groups are presented in Table 2. There were no statistically significant differences between the groups at the beginning of the study, with a predominance of females, single individuals, moderate impairment of functional capacity, minimal depression or dysphoria, and moderate anxiety.

**Table 2:** Sociodemographic characteristics and clinical variables of participants at baseline.

	AS+LSS group (n=17)	PS+LSS group (n=17)
Age (years)	24.53 (6.22)	27.35 (9.44)
Weight (kg)	65.16 (12.84)	66.60 (12,99)
Height (cm)	1.65 (0.07)	1.66 (0.07)
Body mass index (kg/m^2^)	22.50 (12.84)	28.18 (5.81)
Gender (n, % - female/male)	13 (76.47%) / 4 (23.53%)	15 (88.23%) / 2 (11.76%)
Marital status (n,%)		
Married	4 (23.53%)	3 (17.64%)
Single	12 (70.58%)	14 (82.35%)
Divorced	1 (5.88%)	0 (0.00%)
Education Level (n,%)		
Undergraduate university	2 (11.76%)	6 (35.29%)
Incomplete higher education	15 (88.23%)	9 (52.94%)
High school	0 (0.00%)	1 (5.88%)
Incomplete primary education	0 (0.00%)	1 (5.88%)
Numeric Pain Rating Scale (0-10)	5.70 (1.50)	5.90 (1.40)
Total Pain McGill questionnaire (0-67)	28.60 (12.20)	27.20 (5.70)
Sensory McGill questionnaire (0-34)	16.10 (5.90)	14.70 (0.70)
Affective McGill questionnaire (0-17)	4.90 (4.20)	4.10 (4.20)
Roland-Morris disability questionnaire (0-24)	8.10 (4.00)	7.60 (0.00)
Beck depression inventory (0-63)	10.60 (8.10)	10.80 (9.20)
Global Anxiety-Visual Analog Scale (GA-VAS)	6.20 (2.80)	6.30 (1.40)

Categorical variables are reported as n (%) and continuous variables as mean±SD. There were no statistically significant differences at baseline (independent Student's *t*-test and chi-squared test). AS+LSS, active trunk stretching plus lumbar segmental stabilization; PS+LSS, placebo stretching plus lumbar segmental stabilization.

### Numeric pain rating scale (NPRS) and McGill pain questionnaire (MPQ)

**Figure 2:**
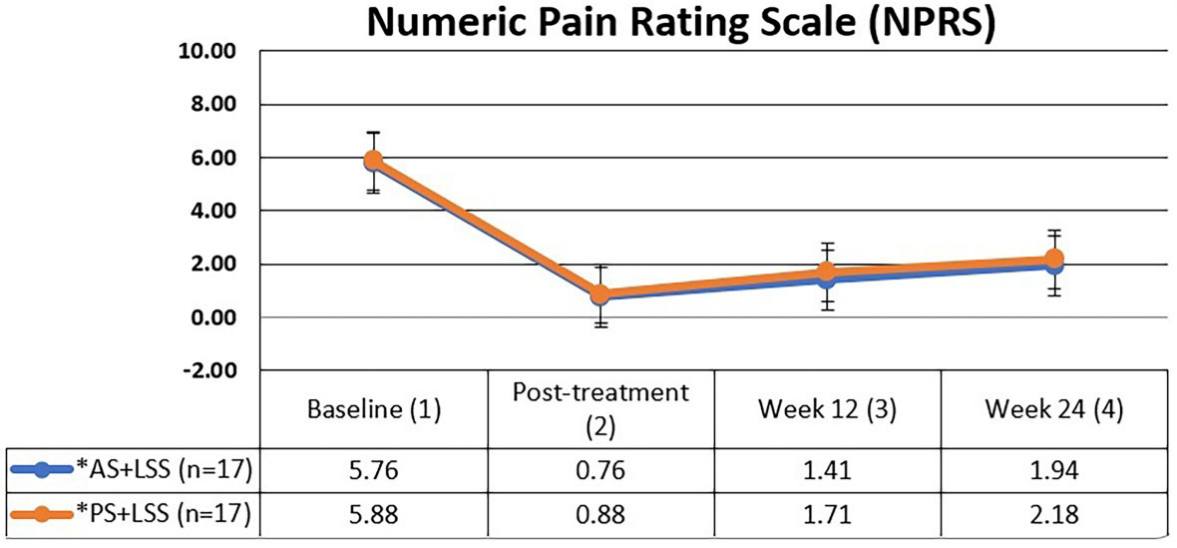
Mean and standard deviation of pain intensity values (NPRS) over time for both groups. The Mann-Whitney test was used for intergroup comparison (P=0.869) and the Friedman test (Friedman's multiple comparisons) was used for intragroup comparison. P<0.001 between 1 and 2, 1 and 3, and 1 and 4 time points for both groups. AS+LSS: active trunk stretching + lumbar segmental stabilization group; PS+LSS: placebo stretching + lumbar segmental stabilization group.

**Figure 3:**
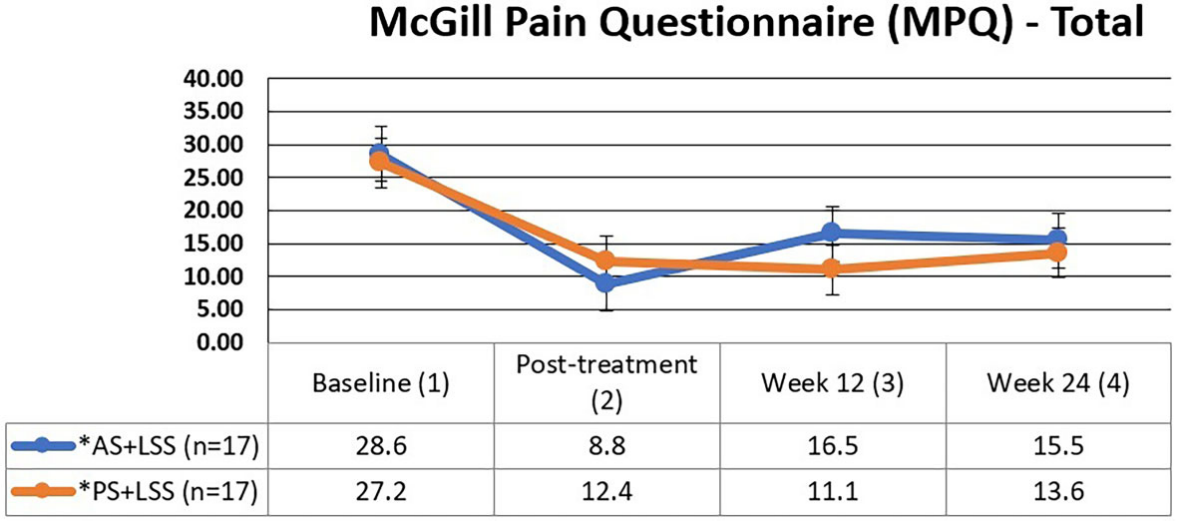
Mean and standard deviation of pain intensity and quality values (MPQ) at baseline (1), post-treatment (2), week 12 (3), and week 24 (4) for both groups. Intergroup comparison was performed using the Mann-Whitney test (P=0.769). The Friedman test (Friedman's multiple comparisons) was used for intragroup comparison. P<0.001 between 1 and 2, 1 and 3, and 1 and 4 time points for both groups. AS+LSS: active trunk stretching + lumbar segmental stabilization group; PS+LSS: placebo stretching + lumbar segmental stabilization group.

There were no statistically significant differences observed between the groups in terms of pain intensity (NPRS) (Figure 2) and pain quality (MPQ) (Figure 3). However, intragroup analyses unveiled a noteworthy trend: both treatment modalities showcased substantial reductions in pain intensity and improvements in pain quality (sensory, affective, and mixed dimensions) post-treatment, persisting at the three and six month follow-up (Table 3).

**Table 3:** Comparison of intra-group and inter-group differences in pain intensity, functional limitation, depression, and anxiety between baseline and at discharge (6 weeks), 12 weeks, and 24 weeks post-discharge.

Weeks	Groups	Numeric Pain Rating Scale	McGill Pain Questionnaire	Rolland-Morris Disability Questionnaire	Beck Depression Inventory	Global Anxiety-Visual Analog Scale
6 - baseline	AS+LSS	**-5.00** **(-6.14 to -3.86)**	**-19.77** **(-29.89 to -9.64)**	**-4.53** **(-6.46 to -2.60)**	**-4.17** **(-7.66 to -0.69)**	-1.65(-3.38 to 0.09)
	PS+LSS	**-5.00** **(-6.14 to -3.86)**	**-14.88** **(-25.01 to -4.76)**	**-4.77** **(-6.69 to -2.84)**	-2.65(-6.13 to 0.83)	-1.71(-3.44 to 0.03)
12 - baseline	AS+LSS	**-4.35** **(-5.70 to -3.01)**	**-12.12** **(-20.68 to -3.55)**	**-5.12** **(-7.27 to -2.97)**	-4.35(-8.95 to 0.24)	-1.71(-3.52 to 0.11)
	PS+LSS	**-4.18** **(-5.53 to -2.83)**	**-16.18** **(-24.74 to -7.61)**	**-5.77** **(-7.92 to -3.61)**	-3.77(-8.35 to 0.83)	-1.41(-3.23 to 0.40)
24 - baseline	AS+LSS	**-3.82** **(-5.26 to -2.39)**	**-13.12** **(-22.08 to -4.15)**	**-5.88** **(-8.12 to -3.65)**	-1.65(-7.36 to 4.07)	-0.53(-2.36 to 1.30)
	PS+LSS	**-3.71** **(-5.15 to -2.27)**	**-13.65** **(-22.61 to -4.68)**	**-4.88** **(-7.12 to -2.65)**	-4.00(-9.72 to 1.72)	-1.00(-2.83 to 0.83)
6 - baseline	AS+LSS *vs* PS+LSS	-0.12(-1.24 to 0.99)	-3.59(-12.61 to 5.42)	0.68(-1.42 to 2.78)	-1.50(-6.49 to 3.49)	-0.05(-1.89 to 1.79)
12 - baseline	AS+LSS *vs* PS+LSS	-0.29(-1.41 to 0.82)	5.35(-3.66 to 14.36)	1.09(-1.01 to 3.19)	-0.56(-5.55 to 4.43)	-0.39(-2.24 to 1.44)
24 - baseline	AS+LSS *vs* PS+LSS	-0.24(-0.87 to 1.35)	1.82(-7.19 to 10.83)	-0.55(-1.55 to 2.66)	2.38(-2.61 to 7.38)	-0.37(-1.48 to 2.21)

Data are reported as mean difference and 95%CI, obtained from mixed linear models (group, time, and interaction; AR(1) covariance). Data in bold denote statistical significance. AS+LSS: Active Trunk Stretching + Lumbar Segmental Stabilization group; PS+LSS: Placebo Stretching + Lumbar Segmental Stabilization group.

The relative gain was remarkable for both the AS+LSS (87%) and PS+LSS (85%) groups, primarily assessed via the Visual Analog Scale (VAS), compared to the MPQ (AS+LSS-69%; PS+LSS-55%) over the follow-up period.

### Roland-Morris disability questionnaire (RMDQ)

**Figure 4:**
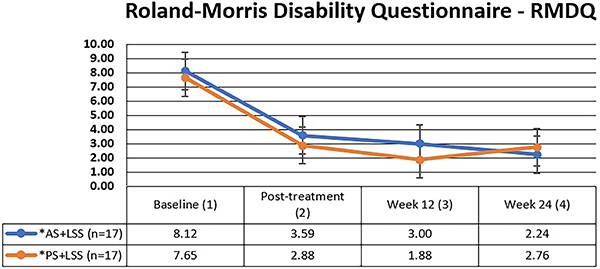
Mean and standard deviation of functional disability values (RMDQ) over time for both groups. The Mann-Whitney test was used for intergroup comparison (P=0.411) and the Friedman test (Friedman's multiple comparisons) was used for intragroup comparison. P<0.001 between 1 and 2, 1 and 3, and 1 and 4 time points for both groups. AS+LSS: active trunk stretching + lumbar segmental stabilization group; PS+LSS: placebo stretching + lumbar segmental stabilization group.

No significant differences in functional capacity were observed between the groups at baseline, post-treatment, and at 3 and 6 months, as assessed by the RMDQ (Figure 4). Both interventions demonstrated improvement from baseline to the end of the period, with initial values indicating moderate disability and transitioning to minimal disability by the end of the (Table 3).

### Beck depression inventory (BDI)

**Figure 5:**
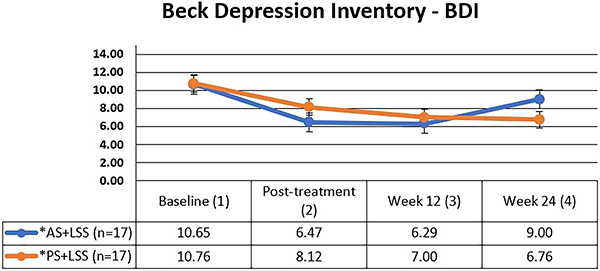
Mean and standard deviation of depression values (BDI) over time for both groups. The Mann-Whitney test was used for intergroup comparison (P=0.265) and the Friedman test (Friedman's multiple comparisons) was used for intragroup comparison. P<0.001 between 1 and 2, 1 and 3, and 1 and 4 time points for both groups. AS+LSS: active trunk stretching + lumbar segmental stabilization group; PS+LSS: placebo stretching + lumbar segmental stabilization group.

No significant differences in symptoms of depression were observed between the groups at baseline, post-treatment, and at 3 and 6 months (Figure 5). The symptoms of depression exhibited a significant reduction during the intervention in both groups, transitioning from minimal depression or dysphoria to mild depression post-treatment. However, this mild level was sustained only in the AS+LSS group upon discharge (MD=-4.17; 95%CI=-7.66 to -0.69) (Table 3).

### Global anxiety-visual analog scale (GA-VAS)

Both groups showed improvement during the treatment period (P<0.006), but there was no significant difference between baseline and the six month follow-up (P=0.809).

Regarding the GPE scale, intragroup analysis did not reveal significant differences. However, intergroup analysis showed that the AS+LSS group improved by 1.19 points (95%CI=0.32 to 2.05) compared to the PS+LSS group after 24 weeks of follow-up (Table 3).

## Discussion

This study did not observe a statistically significant difference between the AS+LSS and PS+LSS groups regarding the treatment effects on the studied variables. However, both groups demonstrated a considerable reduction in pain levels (both intensity and quality) and functional disability immediately post-treatment, with a clinically significant relative gain. Importantly, this relative gain was sustained at 12 and 24 weeks post-treatment for both groups, indicating the efficacy of the treatments in preventing the recurrence of low back pain (31).

Our study hypothesized that trunk stretching combined with LSS could enhance the benefits of pain reduction, functional disability improvement, and emotional aspects among patients with CNLBP. This hypothesis was based on the premise that stretching increases joint space, reduces pressure on the intervertebral disc, and dissipates tension. However, our findings did not find an effect of adding trunk stretching to LSS exercises, as the studied variables did not exhibit additional improvement with this combination. Therefore, the use of LSS alone was sufficient for reducing pain and functional disability in patients with CNLBP and may serve as an effective intervention in clinical practice.

Although our sample size calculation was conducted with methodological rigor to ensure 80% power, it is important to consider that the study may still have been underpowered to detect small between-group differences, particularly given the outcome variability typically observed in CNLBP. Thus, the absence of statistically significant differences may be related to a type II error, as discussed in trials with moderate sample sizes (32). Nonetheless, both groups achieved clinically meaningful improvements based on established minimal important differences for NPRS, RMDQ, BDI, and GPE, reinforcing the clinical relevance and effectiveness of the interventions.

This finding seems to corroborate the recent meta-analysis conducted by Hayden et al. (14), which suggests that all types of exercises, except stretching exercises when adjusted for dose and additional co-interventions, are consistently more effective than minimal care and other treatments for reducing pain and functional limitations in individuals with chronic low back pain.

Our findings are consistent with recent randomized controlled trials (RCTs) that have examined the effects of lumbar segmental stabilization (LSS) and stretching in individuals with CNLBP. Regarding intervention duration and intensity, our protocol adhered to the minimal effective duration of 6 to 8 weeks of supervised sessions, as supported by Hayden et al. (14) and Searle et al. (7). Similarly, Nava-Bringas et al. (33) employed a comparable frequency (twice weekly) and intervention period (six weeks) for LSS and reported significant improvements in pain and disability. However, they found no additional benefit when stretching was combined with stabilization exercises, which aligns with our results.

Likewise, Ahmed et al. (34) examined the effects of dynamic stabilization exercises with and without adjunct manual therapy in individuals with CNLBP and also highlighted improvements in both groups, reinforcing the efficacy of segmental stabilization.

Our study and others, such as those by Nava-Bringas et al. (33) and Lawand et al. (21), adopted longer follow-up periods and more rigorous methodological designs, which enhance the robustness and clinical applicability of the findings.

In terms of patient characteristics, most recent trials, including ours, have focused on adults under 50 years of age, which is consistent with evidence suggesting that younger individuals may respond more favorably to stabilization exercises (35).

Additionally, Lawand et al. (21) demonstrated that stretching alone was beneficial compared to pharmacological management but did not outperform active exercise strategies such as LSS. Although our findings did not show additional effects of stretching when combined with LSS, Lawand et al. (21) reported improvements in pain, function, and quality of life, albeit with no significant impact on depressive symptoms.

Furthermore, as highlighted by Azer et al. (36), stretching remains a widely adopted strategy by patients, emphasizing the need for high-quality clinical trials to establish its true efficacy in the context of multimodal interventions.

Another noteworthy observation pertains to the age distribution of our study participants, which may have influenced the outcomes. There is ongoing discourse regarding the existence of subgroups within CNLBP patients who might derive varied benefits from distinct therapeutic interventions. Notably, the study by Fritz et al. (35) suggests that individuals under the age of 40 tend to benefit from LSS programs. Interestingly, the mean age of the patients enrolled in our study falls within this demographic, thereby reinforcing the alignment with therapeutic recommendations proposed by these authors.

LSS engages deep muscles, such as the lumbar multifidus and transversus abdominis (13), both of which are commonly affected in cases of CNLBP. These exercises modulate pain by triggering the release of β-endorphin, an endogenous opioid involved in pain management (37) while also influencing motor control through the development of strength in deep abdominal muscles. This, in turn, helps mitigate the risk of low back pain episodes. As a result, LSS demonstrates efficacy in reducing recurrent CNLBP episodes over the long term and shows comparable benefits to other active exercise modalities (38).

In our study, we noted immediate post-treatment reductions in depression (assessed by the BDI) and anxiety (measured by the Anxiety Scale). However, these reductions were not maintained throughout the 24-week period, highlighting an important consideration. Elevated anxiety levels and the presence of depression can exacerbate muscular tension and may contribute to, or result from, low back pain. Additionally, the positive impact of the therapeutic alliance between therapist and patient on these variables diminished over the follow-up period. This finding suggests that exclusively physical interventions, such as those tested, may be insufficient to maintain lasting emotional benefits. Cherkin et al. (39) demonstrated that adding mindfulness or cognitive-behavioral therapy to usual care improved pain and function in chronic low back pain, which may support the suggestion of future studies that combine LSS with such approaches.

No adverse effects were reported throughout the intervention period. These findings align with the existing literature, which highlights a low risk of adverse events associated with both LSS and stretching interventions (7,14), reinforcing the safety profile of both approaches. Furthermore, the exercise program utilized in this study demonstrated minimal variability and was straightforward to implement, rendering it highly suitable for adoption by healthcare services in the management of this dysfunction.

A limitation of this study was that adherence to the instruction of avoiding analgesic use and other therapeutic interventions during the study period was not quantitatively recorded. Although participants received verbal instructions, and adherence was monitored through self-reports documented in clinical records by the physical therapist, this approach may be subject to reporting bias. Additionally, despite rigorous blinding and the use of an active control group, we acknowledge that placebo effects and participant expectations may have influenced the outcomes, as commonly observed in chronic pain trials.

## Conclusions

In conclusion, our findings suggest that augmenting LSS exercises with trunk stretching exercises did not yield discernibly superior outcomes for pain alleviation and functional improvement in individuals with CNLBP compared to lumbar segmental stabilization exercises alone. Furthermore, no significant differences were observed in emotional state, anxiety levels, adverse events, patient satisfaction, or treatment perception between the two intervention groups.

## Data Availability

All data generated or analyzed during this study are included in this published article.
